# Oestrogen receptors in breast cancer: basic mechanisms and clinical implications

**DOI:** 10.3332/ecancer.2013.370

**Published:** 2013-11-05

**Authors:** Cecilia Williams, Chin-Yo Lin

**Affiliations:** Center for Nuclear Receptors and Cell Signaling, Department of Biology and Biochemistry, University of Houston, Houston, Texas 77204, USA

**Keywords:** breast cancer, hormonal carcinogenesis, endocrine therapy, oestrogen receptor

## Abstract

Since the discovery of the connection between ovarian hormones and breast cancer, endocrine therapy has been an integral adjuvant treatment for patients with hormone-dependent breast cancers. Oestrogen receptor (ER) plays a central role in mediating the effects of endogenous hormones and therapeutic agents. ER serves as a prognostic marker for responsiveness to endocrine therapy and is targeted either directly by selective oestrogen receptor modulators (SERMs) and pure antagonists or indirectly by aromatase inhibitors (AIs) that block oestrogen production. A significant number of ER-positive patients, however, fail to respond to therapy or develop resistance over time. This review focuses on the current understanding of ER functions and recent advances in genomic technologies and research that have provided a global perspective on hormone and ER activity and led to a number of significant discoveries, including the roles of co-regulatory factors and non-coding RNAs. Mechanistic insights into normal ER functions and therapeutic actions of SERMs and AIs will enable the development of better predictive markers and more effective target mechanisms and ultimately facilitate improvements in disease outcomes and patient survival.

## Introduction

A lady with growth neoplastic

thought castration was just a bit drastic.

She preferred that her ill could be cured with a pill.

Today it’s no longer fantastic.

This quatrain, composed by Elwood Jensen and V. Craig Jordan, amusingly and succinctly summarises one of the great triumphs in breast cancer research and treatment [1]. In 1896, George Beatson reported the beneficial effects of oophorectomy, the female equivalent of castration, in two of his patients with inoperable breast cancer [2]. From his earlier studies of ovulation and lactation, Beatson astutely made the connection between ovarian functions and influences, subsequently shown to be the ovarian hormone oestrogen, with phenotypic changes in mammary tissues and possible link to cancer. He took the first steps in testing this hypothesis, and his seminal discovery provided the first evidence for hormonal carcinogenesis and the potential efficacy of targeting ovarian and hormonal functions. With contributions by Jensen, Jordan, and many others, endocrine therapy, using pills that block oestrogen production or activity, is now routinely applied in the treatment of breast cancer. Other examples of targeted therapy in breast cancer include the use of monoclonal antibodies (trastuzumab) and small molecule receptor tyrosine kinase inhibitors (lapatinib) in targeting the HER2/neu growth factor receptor-positive tumours [3]. This review focuses on the role and mechanisms of action of oestrogen receptors (ERs) in mediating the effects of oestrogen and endocrine therapeutic agents and discusses current challenges and opportunities in targeting ER and oestrogen signalling in the prevention and treatment of breast cancer.

## Discovery and characterisation of ERs

Jensen and Jacobson were the first to observe the retention of radiolabelled oestrogen in hormone-responsive target tissues [4]. Subsequently, work by Jensen, Gorski, and their respective groups demonstrated the existence of intracellular oestrogen-binding receptor proteins [5–8]. The *ER *gene was cloned by the Chambon group, and mutagenesis studies showed that the receptor consists of a DNA-binding domain containing zinc finger motifs and a ligand-binding domain, key structural elements of ligand-dependent transcription factors [9, 10]. Functional studies also identified the N-terminal activating function (AF-1) domain, which is involved in protein–protein interactions important for the transcriptional activity of ER [11]. The identification of other related receptors places ER in the nuclear receptor superfamily of transcriptional regulators [12]. Molecular characterisation of ER revealed that, upon ligand activation, ER regulates target gene expression by binding cis-regulatory elements termed oestrogen response elements (EREs; consensus 5′-GGTCAnnnTGACC-3′). This interaction is facilitated by the pioneering factor FOXA1 [13]. ER can also bind DNA indirectly by tethering to other transcription factors, including AP-1, Sp1, NFκB, and RUNX1. DNA-bound ER nucleates co-regulator complexes that modify chromatin and render the DNA accessible to the transcriptional machinery [14, 15]. ER co-regulators include those that enhance transcriptional activity by altering nucleosome spatial orientation (SWI/SNF) or by modifying histones through acetylation (SRC1, CBP/p300, p/CAF, and p/CIP/AIB1) and methylation (CARM1, PRMT1) [16–25]. Some co-regulators such as NCoR, SMRT, NRIP1, LCoR, and REA function as nuclear receptor co-repressors and play key roles in modulating receptor activity [26–31]. The combination of interactions among ligand, ER, other transcription factors, ERE sequences, differential recruitment of co-regulators, and the overall allosteric effects on receptor complexes allows for an intricate pattern of gene- and tissue-specific effects on target gene expression. In addition to its nuclear functions, ER has been shown to exert rapid non-genomic effects through interactions with cell membrane-associated growth factor receptors and components of downstream signal transduction pathways in the cytoplasm [32]. Post-translational modifications of ER provide additional regulatory mechanisms and enable integration of signals from multiple pathways with oestrogen signalling [33].

Adding to the mechanistic complexity and refinement of oestrogen signalling, a second *ER *gene was discovered in 1996 by Gustafsson and Kuiper and was named ERβ [34]. The original ER was renamed ERα. ERα and ERβ share a 56% similarity in their ligand-binding domains, and both bind the predominant endogenous oestrogen 17β-estradiol. The differences in their ligand-binding domains, however, also result in selective binding of natural and synthetic ligands and allow for selective targeting of each receptor subtype. The two receptors have nearly identical DNA binding domains and share affinity for the canonical ERE. Studies of ERα-positive MCF7 breast cancer cells engineered to express ERβ have confirmed a substantial overlap of DNA-binding sites between the two receptors [35–37]. Intriguingly, their similarities in DNA binding resulted in different gene expression profiles with only a minority of ERβ-regulated genes also regulated by ERα [36, 38–41]. These functional differences may be due to the low conservation of their respective N-terminal AF-1 domains and their different abilities to interact with co-regulators [42]. When co-expressed, ERα and ERβ can function as both homodimers and heterodimers; these complexes appear to have their own transcriptional activities and regulate distinct gene sets [43, 44].

While both receptors are found in the normal breast, ERβ expression appears to be more widespread in mammary tissues [45, 46]. In both the rodent mammary gland and in the human normal breast, ERβ is found in epithelial and stromal cells, while ERα is only expressed in a subset of epithelial cells [46–48]. Nonetheless, ERα is the main mediator of the oestrogen-regulated ductal elongation and growth at puberty and during the menstrual cycle, although this is at least partly a systemic effect through the hypothalamic/pituitary axis [49, 50]. ERβ knockout mice have normal ductal and alveolar development [51], but ERβ is involved in the final terminal differentiation of the mammary gland [47].

ERα is upregulated in the majority of breast cancers, and its expression is a hallmark of hormone-dependent tumour growth. ERβ levels, in contrast, are decreased in tumour cells [52–57]. Whereas ERα is clearly linked to prognosis and response to endocrine therapy, there is no clear evidence that ERβ expression is linked to clinical parameters in breast cancer. This may be due to difficulties in accurately quantifying ERβ protein levels using existing reagents and techniques [58]. While oestrogen treatment of ERα-positive breast cancer cells stimulates proliferation, exogenously introduced ERβ in some studies suppresses ERα-induced proliferation and transcriptional activity while also inducing independent transcriptional and functional changes [40, 41, 59–62]. Related to these anti-proliferative effects, it has also been reported that ERβ-positive tumours may respond more favourably to tamoxifen, and ERβ agonist treatment of ERα-positive breast cancer cell lines appear to enhance their sensitivity to tamoxifen [63, 64]. Re-introduction of ERβ in more invasive ERα-negative breast cancers can, however, increase cell proliferation [65, 66]. The body of data correlating ERβ to both anti-proliferative and proliferative parameters suggests a bifurcated role for ERβ breast cancer biology, but the exact function of ERβ in tumourigenesis and disease progression remains to be determined [66].

## Targeting ER and oestrogen signalling in breast cancer prevention and treatment

For several decades following Beatson’s initial published report, castration by surgical means or by irradiation was used to treat premenopausal women with recurrent or distant metastatic breast cancer. In some postmenopausal women, high doses of androgen or, paradoxically, the synthetic non-steroidal oestrogen diethylstilbestrol was effective in the treatment of advanced diseases [67–69]. Identification of ERα and the development of methodology to detect its expression by hormone binding assays in tumour samples enabled the clinical studies required that ERα be established as a prognostic marker for response to hormone therapy, and determining the ERα-status of tumour samples is now standard practice in clinical oncology [7]. 

The major breakthrough in targeting oestrogen signalling and ERα came from the development of non-steroidal anti-oestrogens using derivatives of triphenylethylenes by the pharmaceutical industry. The goal of these efforts was to develop anti-oestrogenic compounds that can be used in contraception. One compound, ICI 46, 474, had modest effects on fertility but showed promise as an anti-cancer agent with comparable effects with castration or hormone therapy [70]. This compound, later named tamoxifen, was shown to bind ERα, disrupt the binding of oestrogen, and block hormone-dependent breast cancer cell proliferation and tumour formation [71–73]. Following extensive pre-clinical and clinical studies, tamoxifen was approved for the treatment of ERα-positive breast cancers and for the prevention of breast cancer in high-risk individuals.

An early concern regarding the application of anti-oestrogens is their potential impact on the beneficial effects of oestrogen on bone density and cardioprotection. Interestingly, while blocking the effects of oestrogen in breast cancer cells, tamoxifen treatment actually improved bone density and reduced circulating levels of the harmful low-density lipoproteins. One of the negative effects of this selective action is that tamoxifen increases endometrial cell proliferation and risk for endometrial cancers [74]. Another non-steroidal anti-oestrogen candidate, keoxifene, later renamed raloxifene, was demonstrated to be effective in treating osteoporosis and was also approved for the prevention of breast cancer. Compared with tamoxifen, raloxifene does not have an effect on endometrial cell growth and proliferation. Tamoxifen and raloxifene are the first members of a class of drugs, termed selective oestrogen receptor modulators (SERMs). They exhibit both oestrogenic and anti-oestrogenic effects in a tissue-specific manner and raise the possibility of simultaneously targeting multiple endocrine-related diseases or conditions. An alternative approach for directly targeting ER in breast cancer treatment is through the use of pure anti-oestrogens. Fulvestrant, initially designated as ICI 182,780, is a steroidal compound with high affinity for ERα. In addition to blocking ER activity, treatment with fulvestrant also leads to the rapid degradation of ER proteins. Consequently, treatment completely disrupts ER activity, as compared with the SERMs. This drug is particularly effective as second-line treatment when tumour cells develop resistance to tamoxifen but still require ER for continuing proliferation [75].

As the role of oestrogen became apparent in hormonal carcinogenesis and disease progression in the majority of breast cancers, an alternative strategy for targeting oestrogen signalling and ER functions emerged. Aromatase is a key enzyme involved in the conversion of androgen to oestrogen by catalysing the aromatisation of the A ring in testosterone. Inhibition of aromatase activity indirectly targets ER functions by effectively starving hormone-dependent tumour cells of locally produced oestrogens. Steroidal (exemestane) and non-steroidal (anastrozole, letrozole) aromatase inhibitors (AIs) have been developed to selectively target aromatase enzymes. These compounds either bind and inactivate aromatase or compete with endogenous substrates to reduce oestrogen production. In clinical trials, AIs showed improved efficacy as compared with treatments with tamoxifen, and these drugs are now approved for use in the adjuvant therapy of postmenopausal patients with ER-positive tumours [76–78].

## Challenges and opportunities

ERα protein level, as noted previously, is the major marker for potential response to endocrine therapy. Progesterone receptor (PR), an ERα target gene, expression is an additional marker for responsiveness. Not all tumours that are classified as ERα-positive, however, respond to treatments. Resistance to endocrine therapy is estimated at about 40% [79]. The evolutionary history and specific somatic mutations that gave rise to the primary tumours may have rendered them non-responsive prior to diagnosis and subsequent treatment. Moreover, the selective pressures of long-term endocrine treatment may drive the evolution of resistant tumour cells and recurrent tumours. Mechanisms of resistance to endocrine therapy include hypersensitivity to low levels of oestrogen following treatments with AIs, alternative activation of ERα via growth factor-mediated pathways and mechanisms, and complete oestrogen- and ERα-independent growth and proliferation of tumour cells [80]. Another challenge in the application of endocrine therapy is the treatment of premenopausal patients where disruption of hormone production and ER functions may be less effective and desirable and also introduces side effects, which may increase susceptibility to other diseases following long-term treatments [81]. In spite of the benefits of current endocrine therapeutic options, further scientific and technical breakthroughs are required to fully realise the potential of targeting endocrine-related mechanisms and reducing the morbidity and mortality associated with hormone-dependent breast cancers.

Advances in genomics and genomic technologies have contributed significantly to biomedical research in general and provided a number of mechanistic insights into ER biology in breast cancer cells. These insights have resulted in candidate markers and target mechanisms in endocrine therapy. For example, gene expression profiling studies using microarrays have identified hundreds of oestrogen responsive genes, both transcriptional targets as well as those downstream of ER-regulated signalling pathways, which can be exploited as both markers of oestrogen responsiveness in tumour cells and as targetable genes and gene networks, which specifically regulate tumour cell proliferation [82–84]. Comparative analysis of sensitive and resistant cells may further elucidate markers and mechanisms of resistance. Similar gene expression studies in clinical samples have identified gene sets and signatures that define clinical subtypes and predict response to endocrine therapy and may also suggest potential resistant mechanisms [85]. Genome-wide mapping studies of ER binding sites and computational modelling of sequence motifs have identified co-localising transcription factors such as FOXA1, GATA3, and AP-2γ that are required for ER transcriptional regulatory activity and represent additional candidate markers and therapeutic targets [13, 86, 87]. Improvements and innovations in proteomic technologies also contribute to our understanding of the ER complex, including associated co-regulators and transcription factors and may define potential markers and targets [88].

**Figure 1.**
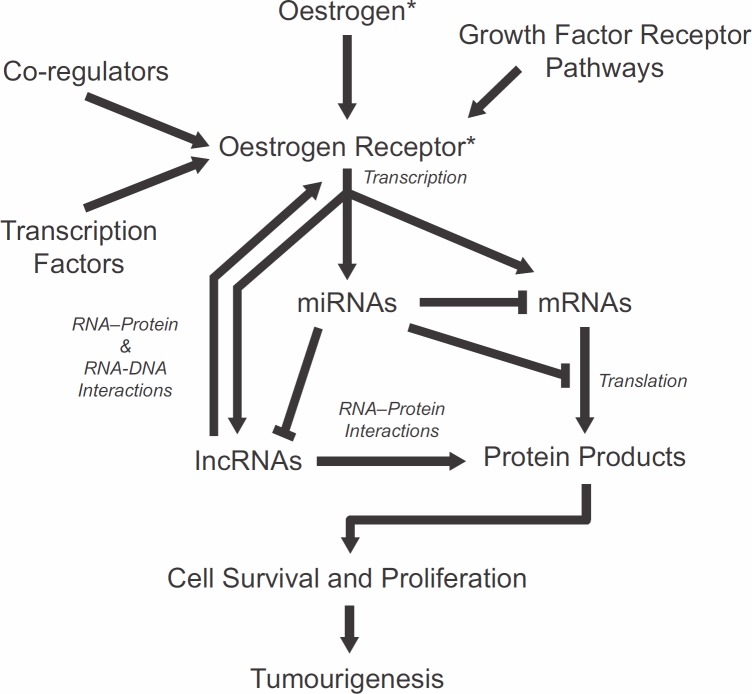
Summary of molecular interactions and mechanisms involved in oestrogen signalling and oestrogen receptor functions. Each component represents potential markers and target mechanisms for endocrine therapy. ^*^Targets of current endocrine therapeutics.

Genomic studies have also highlighted the emerging importance of non-coding RNAs in basic and translational research. Small microRNAs (miRNAs) serve as key regulators of gene expression by targeting genes for degradation or by blocking their translation. ERα-positive breast cancers display a distinct miRNA-expression profile compared with ERα-negative breast cancers [89–92]. Whether ERα directly regulates miRNA is not clear, but miRNA regulations are nonetheless likely to occur indirectly via other oestrogen-responsive genes or through ERα interaction with the miRNA processing machinery [93–95]. In addition, several miRNAs, including miR-206, have been shown to regulate ERα expression by targeting the 3′ untranslated region of its mRNA [96, 97]. Transcriptome-wide nuclear run-on studies have identified long non-coding RNAs (lncRNAs) as early targets of activated ER [98]. These transcripts share the same features as protein-coding RNAs such as capping, splice sites, and polyadenylation but encode extremely short open-reading frames. Functionally, lncRNAs participate in RNA–protein, RNA–RNA, and RNA–DNA interactions in molecular processes, including those that are involved in cancer-related functions [99, 100]. Recent report by Li and colleagues shows that a specific type of lncRNAs transcribed from enhancer regions of ER target genes and named enhancer RNAs, function in the looping of chromatin that facilitates interactions between distal regulatory sites with promoters of target genes [101]. Non-coding RNAs can be specifically targeted by complementary RNAs, and their expression and function disrupted by the cellular RNA interference mechanisms [102]. The rapid progress in understanding the roles of RNAs in oestrogen signalling and ER functions suggests the potential of applying RNA therapeutics, singly or in combination with existing chemo- and endocrine therapy drugs, in improving the specificity and efficacy of endocrine therapy in breast cancer prevention and treatment. Mechanisms of oestrogen signalling and ER action and potential markers and targets are summarised in Figure 1.

## Conclusion

Current successes in the treatment of hormone-dependent breast cancers still leave room for significant improvements in the specificity and efficacy of current endocrine therapeutic approaches and in overcoming resistant tumours. Accumulating insights regarding oestrogen signalling and mechanisms of action of ligands and ER provide opportunities for the development of novel markers, targets, and therapeutic strategies.

## Conflict of interest statement

The authors declare that they have no conflict of interest.
